# Scope and impact of a First Nations cancer coordinator role: perspectives of multidisciplinary cancer staff

**DOI:** 10.1007/s00520-026-10351-4

**Published:** 2026-01-24

**Authors:** Mollie Wilson, Marissa Mulcahy, Jennifer Philip, Brian Le, Sue-Anne McLachlan, Gail Garvey

**Affiliations:** 1 Department of Medicine, University of Melbourne, Melbourne, Australia; 2 Palliative Nexus, St Vincent’s Hospital Melbourne, Melbourne, Australia; 3 The Royal Melbourne Hospital, Melbourne, Australia; 4 Faculty of Medicine, The University of Queensland, Brisbane, Australia

**Keywords:** First Nations, Responsive cancer care, First Nations Cancer Coordinator (FNCC)

## Abstract

**Purpose:**

Aboriginal and Torres Strait Islander people (hereafter respectfully referred to as First Nations) experience poorer cancer outcomes and higher mortality rates compared to non-Indigenous Australians. Barriers in accessing and engaging with cancer care services contribute significantly to these disparities. The First Nations Cancer Coordinator (FNCC) role offers a model that combines cultural and coordination support to improve the navigation of cancer services for First Nations people. This project sought to understand perceptions and impact of a pilot FNCC intervention on the care of First Nations patients, from the perspective of cancer care teams.

**Methods:**

An exploratory, qualitative study was undertaken. The study was co-designed with a First Nations Community Advisory Group. Semi-structured interviews were undertaken with ten non-Indigenous cancer staff who had worked alongside the FNCC to support First Nations patients with cancer and their families.

**Results:**

Analysis revealed three overarching themes relating to the scope and impact of the FNCC role: 1) Integrating cultural wisdom in cancer care delivery; 2) Relationship building through dedicated time and presence; 3) Bridging cultures by facilitating two-way communication and trust between patients and healthcare providers.

**Conclusion:**

The findings highlight the critical importance of FNCC roles in advocating for and enhancing cultural safety in cancer care, essential for addressing disparities and improving cancer outcomes for First Nations Australians.

## Background

Aboriginal and Torres Strait Islander Peoples in Australia (hereafter, respectfully referred to as First Nations) experience persistently poorer cancer outcomes, including delayed diagnoses and higher mortality rates [1], compared to non-Indigenous Australians. These disparities are partially due to barriers relating to cancer screening [2, 3] and access to culturally safe and responsive cancer care services [4–7]. First Nations Peoples have reported fear and mistrust of mainstream healthcare services based on historic and recent past [8], experiences of racism [9], healthcare staff with limited cultural understanding [10], being away from Country (Indigenous lands) [11], financial concerns [12], and cultural beliefs and stigma surrounding cancer as barriers to cancer services and care. [13, 14] Miscommunication and a lack of care coordination within, and between services may also create significant barriers to engagement with services and timely, integrated and culturally safe care for First Nations patients [11, 15].

Interventions that are led by and informed by the needs of First Nations Peoples are urgently needed to address cancer inequalities and to promote resilient and culturally grounded approaches to health and wellbeing. One such intervention, supported by First Nations Peoples with cancer in Australia and internationally, is the implementation of First Nations Cancer Coordinator (FNCC) roles (also known internationally as Indigenous Patient Navigators [IPN]) [9, 16–18]. Cancer coordination or patient navigation is a patient-centric healthcare service delivery model that aims to eliminate barriers to effective care and facilitate the timely movement of patients through complicated and often disconnected systems of care [19, 20].

There has been a marked growth in the development of First Nations-led cancer coordination programs in the past decade, with 16 such programs identified in 2022 across USA, Canada and New Zealand demonstrating improved adherence to screening and reduced treatment for those patients accessing IPN/FNCC support [21, 22]. In Australia, IPN/FNCC programs have been piloted in Queensland, New South Wales, South Australia, Western Australia, and, most recently, Victoria. However, the data detailing the impact or the effective implementation strategies of these roles in Australia has not been published.

### Methods

This qualitative study describes the perceptions and impact of the FNCC intervention on the care of First Nations patients, from the perspective of cancer care teams in two metropolitan hospitals.

### Design

This study utilised an exploratory, qualitative design. A co-design methodology was used, underpinned by key principles and best practices of co-design with First Nations Australians [23, 24]. First Nations governance was established through an ongoing partnership with a Community Advisory Group of First Nations Health workers, representatives from local First Nations community-controlled health organisations, senior First Nations researchers and First Nations people with lived experience of cancer. The Community Advisory Group’s collective leadership ensured all aspects of the research process and outcomes upheld First Nations self-determination, were culturally grounded, and more likely to increase the capacity of health systems to meet the needs of the First Nations community.

The COREQ checklist for reporting qualitative research and the CONSIDER checklist for research involving First Nations People were used to guide the reporting of this study [25, 26].

### Setting

In 2023, overseen by the Community Advisory Group, a best-practice FNCC role [27] was developed and informed by literature reviews and guidance from previous IPN programs [28]. The FNCC was a qualified First Nations health practitioner who had extensive experience in First Nations health. As part of the project additional short courses in cancer care were undertaken alongside cancer clinician mentorship relationships established. In addition, throughout the project, the FNCC had access to clinical supervision with a senior palliative care clinician and cultural supervision through the First Nations Health Unit. The FNCC role was piloted at two metropolitan hospital sites in Melbourne, Australia with continuous role development informed by iterative feedback throughout. The role worked collaboratively with First Nations Hospital Liaison Officers and multi-disciplinary cancer teams to support mutual patients [27]. During the study period, the FNCC provided support to 56 patients with cancer, who attended screening or received treatment at the hospital sites.

### Participants

Eligible participants included nurses, physicians and allied health professionals at the participating hospitals, who had worked alongside the FNCC role providing care for First Nations patients with cancer. Participants were purposively sampled to include a range of clinical disciplines, including oncology, general medicine, and psychology. Twelve eligible participants received email invitations to participate in interviews, and those interested provided informed consent.

### Data collection

Between June and July 2024, Zoom interviews were conducted by a non-Indigenous investigator experienced in qualitative research (MW). Two investigators (JP, BL) had a pre-existing, collegial relationship with some participants, which may have facilitated trust and open communication during data collection. All interviews were audio-recorded and transcribed verbatim, with transcripts checked against the recordings for accuracy. Deidentification of transcripts occurred prior to analysis.

Semi-structured interview guides were informed by existing literature and developed through consultation with the Community Advisory Group. Questions were designed to elicit participants’ perceptions of the FNCC role (e.g. “what would you say are the core components of a FNCC role?”) and the impact of the role on the delivery of cancer care (e.g. “can you describe how the FNCC role may have been helpful in the work of your team and/or organisation?”) To safeguard the identity of the FNCC and patients, participants were prompted to share their perceptions of the role in broad, conceptual terms rather than through personal anecdotes. Interviews explored how the FNCC role assisted or interfaced with the existing operations of the cancer teams and the wider hospital/organisation in the delivery of cancer care for First Nations patients. Demographic details were collected using a short, study specific questionnaire, submitted online prior to the interview.

### Data analysis

The data were analyzed using an inductive thematic approach, allowing for broad themes to emerge directly from the data [29, 30]. Initial familiarization with the data was undertaken by the first author (MW), with repeated listening to audio recordings, reading of transcripts, notetaking, and generation of initial codes. The codes were refined and then collated into potential themes, through regular discussions and re-reading with two senior investigators (GG and JP). Ongoing analysis refined the specifics of each theme. Finally, the themes, subthemes and summaries were reviewed and verified by co-authors and the Community Advisory Group.

Our team acknowledges the importance of reflexively considering and describing our own backgrounds, perspectives and values, that we each contribute to the project [31, 32]. MW is a non-Indigenous palliative care researcher, JP is a non-Indigenous palliative care physician and researcher, MM is a First Nations health professional, SM is a non-Indigenous oncologist and researcher, BL is a non-Indigenous palliative care physician and researcher, and GG is a senior First Nations researcher.

### Ethics

Ethics approval was obtained from the Human Research Ethics Committee, St Vincent's Hospital Melbourne (Reference: HREC 250/23). The project was conducted in accordance with the National Health and Medical Research Council’s (NHMRC) ethics and values for research involving First Nations communities [33–35].

## Results

### Participant characteristics

Ten health professionals (six female, four male) participated in interviews (20–50 minutes). Participants included physicians (60%), nurse coordinators (20%), and allied health workers (20%). Most participants (n=8) had more than 10 years of experience in healthcare, and all participants reported experience in providing care to First Nations patients.

### Overview of findings

Analysis of the interviews revealed three overarching themes: 1. Integrating cultural wisdom in cancer care delivery; 2. Relationship building through dedicated time and presence; 3. Bridging cultures by facilitating two-way communication and trust between patients and healthcare providers. Each theme is described according to participants’ perceptions of the FNCC role, and the corresponding impact of these perceptions. Exemplar quotes are included in Table 1.

**Table 1:** Summary of themes and illustrative quotes

Perceptions of the FNCC role		Impact of the FNCC role	
Theme 1. Integrating cultural wisdom in cancer care delivery			
**Sub-theme**	**Illustrative Quote**	**Sub-theme**	**Illustrative Quote**
Embedded member of the multidisciplinary cancer team	“With the [FNCC role], you've got that added extra person that can play the lens of culturally appropriate practice. So, you know, in my mind, they're just another member of the team that have a specialty focus with that group.” (Staff 06)	Presence of role improves the cultural competency of the non-Indigenous cancer workforce	"It's reassuring and best patient practice and care, knowing that there's someone who... has that experience culturally and can go from the perspective of the patient and their cultural needs and make sure that that we are approaching the situation appropriately. That we're providing the care that the person needs and can build those relationships... in a much more meaningful way. I think it's very reassuring to know that.” (Staff 10)
Cultural education and advocacy beyond patient care	“We weren't taught these things at university, that people would have different reasons why they wouldn't feel comfortable to be in hospital and seeking health care. And so having that awareness... that we all have different reasons for why we do or don't seek health care and how we might help support that person.” (Staff 04)	Cultural support at end-of-life	“And my brain immediately goes towards end-of-life goals, comfort, care. So, I'm thinking of things about, you know, spiritual care and being able to actually go back to country is one that I would imagine. Or having family be able to access and support them if they are half a state away, you know.” (Staff 06)
Theme 2. Relationship and trust building with patients and family, through dedicated time and presence			
**Sub-theme**	**Illustrative Quote**	**Sub-theme**	**Illustrative Quote**
Continuity of care and trusted relationships	"And I know that there's a few patients that we've linked in with, and [the FNCC] has more of a role basically in their ongoing care. Like they'll catch up with [the FNCC] more so than me... We communicate if there's things that I need to know or if [the FNCC] needs to know, and it helps to just provide a better outcome for the patients.” (Staff 04)	Central point of contact for patients and families	“Between the doctors and the allied health team, there's lots of different people involved with the patient, and to have a sort of a central point of contact that the family know and are familiar with, and have that ongoing relationship with (the FNCC)... I think that's incredibly valuable.” (Staff 10)
		Person- and family-centered care is cultivated through trusted relationships	“[The FNCC] is going to be able to give us the guidelines that will work best to help a person, and we're able to kind of tell the rest of the team, this is what we're hearing, and this is how we need to work with this person.” (Staff 06)
Theme 3. Bridging Cultures – facilitating connection, two-way communication and trust between patients and healthcare providers			
**Sub-theme**	**Illustrative Quote**	**Sub-theme**	**Illustrative Quote**
Culturally informed coordination between hospital and community services	“[The FNCC] links in with things that I have no idea (exist). The supports that are available once they're outside the hospital... A lot of the time, we give so much here, and then it's like, later, goodbye. So having that extra external [support] is great.” (Staff 05)	Improving trust and engagement with cancer care providers	"So that's what I see roles like the FNCC, that people then feel they have a connection and feel comfortable. And then [the FNCC] can introduce and say, "look, [this Doctor] is really helpful”... And then we can hopefully help provide some care. So that's where I found that was really helpful for me in sort of shaping my understanding of how we might provide more care and better care for people.” (Staff 04)
Trusted conduits between patients and healthcare providers	“So from our side it might be about making sure that they know about their appointments, they get to their appointments, but from their side, that they've got someone that they can communicate with when they've got concerns, who can help to, you know, then liaise with the appropriate people.” (Staff 01)	Streamlining care pathways	"I feel like it is a massive time saving exercise... it's hard to measure what the adverse consequences of not having a FNCC would be. But all the time that's wasted... even if you think of the reduced rapport between the patient and the family members that might otherwise be obtained if there wasn't a specific FNCC helping out in these specific cases. That's going to increase potentially duration of stay... Because you need to get consent for procedures and engagement and rehab, and all these sorts of things, and so the FNCC, in my mind, can support all of the above, and try to convey the need for those things in a way that’s understandable to the patient, which makes them more likely to be motivated and engaged in... medical treatments, therapy supports, whatever is needed.” (Staff 10)

1. *Integrating cultural wisdom in cancer care deliver*

The FNCC was widely accepted as an integral member of the multidisciplinary cancer team, who provided advocacy for culturally appropriate patient care. The addition of the role was reported as an enormous benefit to cultural safety, with Staff 07 reflecting that the FNCC’s “*presence is, in my view, almost as important as what they actually do*.” This statement suggests that the existence of the FNCC position could send a powerful message to First Nations Peoples, signaling that their cultural needs are recognised, valued and central to their care.

a *Perceptions of health care professionals – Embedded member of the multidisciplinary cancer team*The FNCC was viewed as a trusted cultural resource and embedded member of the multidisciplinary cancer team. Participants recognised that the FNCC had unique cultural insights and a deep understanding of the needs and experiences of First Nations patients, equipping them to inform the broader team on the delivery of culturally appropriate, person-centered care. The collegial relationship between FNCC and other staff members facilitated open conversations and a safe platform for non-Indigenous staff members to seek guidance on cultural aspects of patient care. Staff 06 described the FNCC as a cultural “*check and balance*” to ensure that non-Indigenous staff were not basing care on harmful assumptions

Participants acknowledged that the FNCC brought a unique perspective that blended understanding of optimal clinical care standards with a deep knowledge of patients’ cultural needs. This allowed the FNCC to contribute to resources and care plans that integrated medical and cultural aspects in a way that resonated with patients and families. One example of this was described when the FNCC worked alongside a clinician and family members to create a culturally appropriate document that a patient could utilise to keep track of their diagnosis and treatment.

b *Perceptions of health care professionals – Cultural education and advocacy beyond patient care*The FNCC was perceived as a cultural educator and advocate, not only for individual patients but also for the broader healthcare workforce and community. Even without shared patients, the FNCC continued to attend multidisciplinary cancer team meetings and present at in-service meetings, educating staff on cultural, barriers experienced by First Nations Peoples when accessing healthcare services, and First Nations history, culture and significant events.

iii. *Impact – Presence of role improves the cultural competency of the non-Indigenous cancer workforce*Participants reflected that the presence of the FNCC role within the multidisciplinary cancer teams had improved their own cultural competency and understanding of First Nations culture more broadly. The ongoing cultural support enabled non-Indigenous staff to proactively identify and address cultural needs themselves, increasing their confidence in initiating conversations about cultural identity and providing culturally responsive care.

Many participants reported that since working alongside the FNCC, they no longer felt uncomfortable ‘*Asking the Question*’ to establish First Nations status. In part, this was due to increased cultural understanding, but in some interviews, participants remarked that if they were caring for First Nations patients, it was reassuring to know that they could offer them additional, cancer-specific cultural support. Reflecting on staff members’ potential hesitancy to ask the question, Staff 07 suggested that non-Indigenous staff may be worried about what comes next, after First Nations status has been established: “*but if what comes next is - ‘there’s a coordination person in this role, would you like to meet them?*’ - *that makes the conversation much more natural.*”

iv. *Impact – Cultural support at end-of-life. *Participants noted that the FNCC’s guidance was particularly valuable during end-of-life care for First Nations patients, when there were often additional coordination requirements. The FNCC was also able to advocate for and coordinate culturally appropriate practices, both within hospital settings and in supporting patients’ desires to return to Community and Country. In the hospital setting, participants mentioned instances where the FNCC had facilitated the accommodation of extended family and advocated for cultural practices (e.g. smoking ceremonies) that required adjustments to usual hospital policies. Beyond the hospital, the FNCC supported palliative care and other community-based teams to coordinate appropriate care and resource allocation in the patient’s preferred location.

2. *Relationship and trust building with patients and family, through dedicated time and presence*

Participants acknowledged that First Nations Peoples had a range of reasons to distrust the healthcare system, including experiences of racism, the negative experiences of family or community members, and historical and current policymaking that denied the consent and involvement of First Nations Peoples. To improve the credibility and trustworthiness of mainstream medical services, genuine relationship building, between hospitals and community-based services and between healthcare professionals and their individual patients, is essential. Culturally informed roles, such as the FNCC, could incorporate flexibility and a broader scope than usually existing within the Western healthcare model to build meaningful relationships and improve overall trust.

a *Perceptions of health care professionals – Continuity of care and trusted relationships. *Unlike many healthcare roles, typically confined to one treatment stage or care setting, the FNCC could support patients throughout their illness course, both within and outside of the hospital setting. Participants noted that the FNCC had more regular contact with patients and family than other members of the care team: “*My role is more before surgery and things, whereas the [FNCC] role, they can catch up with the patient more after appointments and things as well*” (Staff 05). This unique scope allowed the FNCC to nurture trusted bonds and rapport with patients and families. Other participants described the paucity of time in a typical clinical setting, noting how challenging it could be to “*get to know someone*” within the constraints of a one-off 15-minute consultation. Ongoing care was described as a significant strength of the FNCC role. The FNCC was available to attend clinics and appointments, but also to sit and yarn (culturally safe conversation) with patients in a casual context, provide support in community settings, and contact over the phone. Participants expressed gratitude for the FNCC’s additional capacity, and an awareness that genuine relationship building is vital to ensure trust and comfort in mainstream healthcare services.

b *Impact - Central point of contact for patients and families. *Consistent presence and availability made the FNCC a reliable point of contact for patients and families as they navigated the complexities of what can be a fragmented cancer care system. Participants reflected on the many services and healthcare professionals involved in the provision of cancer care, noting that a single, trusted point of contact may alleviate additional navigation stresses. If this point of contact was known and trusted in the community, this may encourage earlier and timely engagement with cancer services. As one participant described “*for patients, knowing that there is already a person that they know and that they're familiar with, and is that link, that... point of contact. When's my appointment? When am I going? How am I gonna (sic) get there*?... *All those really practical things would be quite helpful*” (Staff 09).

iii. *Impact – Person- and family-centered care is cultivated through trusted and meaningful relationships. *Through trusted relationships fostered over time, the FNCC could gain a nuanced understanding of each patient’s individual needs. This insight allowed the FNCC to guide the broader cancer care team in tailoring approaches that best resonated with patients and their families. For example, participants recalled seeking the FNCC’s advice on the involvement of family and community members in family meetings and other consultations. The FNCC could provide a safe space for patients to voice their concerns, ask questions and engage in open dialogue with their care teams. The FNCC role, as one participant described, “*helps to make our patients stronger on their own, to be able to advocate for themselves*” (Staff 05). The empowering influence of the FNCC went beyond cancer care. One participant described a patient who had never identified with their First Nations heritage throughout previous healthcare interactions, but through the FNCC’s cultural support and knowledge, found an opportunity to explore their cultural background.

3. *Bridging Cultures – facilitating connection, two-way communication and trust between patients and healthcare providers*

As both an embedded member of the cancer team and a trusted point of contact for patients, the FNCC facilitated two-way communication between patients, families and healthcare providers.

a *Perceptions of health care professionals – Culturally informed coordination between hospital and community services. *Participants noted that the FNCC’s cultural knowledge, coupled with their familiarity with cancer pathways, made them well-positioned to provide comprehensive coordination and advocacy for patients, ensuring that patients were connected to available support services. A key linkage that the FNCC could provide was access to local First Nations community-controlled health organisations and cultural support services. These linkages were particularly crucial in facilitating timely access to care prior to diagnosis, and ensuring continuity of care in the community, after discharge from the hospital. Participants noted the large number of rural patients who attend metro hospitals to receive treatment, and their often-significant coordination requirements. The FNCC, through linkages with community services, could help identify patients early and provide support prior to hospital presentation

b *Perceptions of health care professionals – Trusted conduits between patients and healthcare providers. *In addition to being a central point of contact for patients and family members, participants considered the FNCC to serve a similar role within care teams. The FNCC could convey important updates on patients’ needs and situations to the broader healthcare team and community-based services, while simultaneously, communicate critical information about care plans, appointments and treatment options back to patients and families. By facilitating this bidirectional flow of information, the FNCC could ensure that all parties remained informed and ‘on the same page’. Participants found that the FNCC was often able to communicate nuanced information that provided further context to medical record documentation.

iii. *Impact – Improving trust and engagement with cancer care providers. *The strong relationships and rapport with patients and families, coupled with the FNCC’s embedded role within the multidisciplinary team, served as a foundation for trust-building with the broader team. When the FNCC could facilitate introductions or attend initial appointments, participants felt that patients were more at ease and trusting in other healthcare providers. Participants valued the FNCC’s ability to ‘vouch’ for them and reassure patients of their competence and commitment to their wellbeing.

iv. The FNCC’s ability to check in with patients, during and beyond appointments, ensured that they were not overwhelmed or confused by the information that they were receiving. One participant described how challenging it could be to achieve the ‘*right amount’* of information; “*between information... and the information just starting to become white noise, and, worse than that, actually a barrier to what needs to be done*” (Staff 07). Participants reflected that the FNCC’s presence could foster a safe environment of understanding, enabling patients to make informed decisions and exercise self-determination over their cancer care. In one example, a participant described a patient with a complicated history of trauma, who felt intense discomfort about staying in hospital. The patient, who would repeatedly present to the emergency department, “*would only stay because of the involvement of the FNCC*” (Staff 04).

e *Impact – Streamlining care pathways. *As a trusted point of contact, participants felt that the FNCC role reduced potential delays that might arise from multiple points of contact within a fragmented cancer care system, thus enhancing the overall efficiency and timeliness of care. Participants listed a number of ways that the FNCC could save time including improving rapport between patients and healthcare teams, ‘de-medicalizing’ information, conveying the importance of certain treatments or pathways to patients and supporting healthcare teams with consent processes for various procedures. As an embedded member of the cancer team, the FNCC was well-positioned to contact the relevant people when urgent coordination was required.

## Discussion

In Australia, many First Nations Peoples continue to face barriers in accessing culturally safe cancer care. The present study has demonstrated that FNCC roles can address some of these barriers by providing culturally grounded care coordination, facilitating trust between patients and healthcare teams, while simultaneously improving the cultural competency of healthcare workers themselves. These findings contribute to a growing international evidence base that supports the positive impact of IPNs and FNCC roles on the provision of cancer care. [22] Importantly, this is the first study to document the qualitative impact of a FNCC role from the perspectives of non-Indigenous cancer staff.

These findings support existing research that patient navigators operate at the interface between the health system and underserved communities, thus requiring an “insider” knowledge of both [9, 36]. The FNCC should have knowledge of clinical standards and cancer care pathways, in addition to cultural understanding, to operate effectively in both settings [27]. With regards to the former, future FNCC programs should ensure that FNCC’s receive adequate, cancer-specific training, have access to clinical and cultural supervision, and are well connected with multidisciplinary cancer teams. With regards to the latter, it is recommended that FNCC roles be designated specifically for First Nations peoples. Our findings indicate that the FNCC’s cultural standing afforded a unique insight into the needs and concerns of First Nations patients. This supports previous research that First Nations people feel less guarded and more comfortable asking questions when a First Nations staff member is present [11, 37]. Additionally, First Nations identified roles have been found to improve the communication between patients and non-Indigenous healthcare providers [38, 39]. It is the combination of cultural knowledge and capacity for relationship building that makes the FNCC role distinct from existing cancer coordinator positions.

While this study supports evidence that First Nations health workers enhance cultural awareness [38, 39], the responsibility for cultural safety should extend beyond these roles, to all staff and encompass the entire organisation. As Panozzo et al., (2023) described in a palliative care context, there can be an overreliance on First Nations health workers to deescalate communication breakdowns or misunderstandings relating to cultural aspects of care [39]. Health care professionals may assume that it’s the First Nations patients who are unable to express their needs, rather than deepening their own cultural awareness and capacity to provide trauma-informed care [40]. The collaborative nature of the FNCC role, in addition to its broad scope and focus on proactive, early involvement with patients, highlights the positive impact of ongoing cultural support on overall care delivery. Non-Indigenous staff frequently reflected on their increased confidence, interest and understanding in the cultural aspects of care. The collaborative and educational aspects of the FNCC role ensure that cultural safety is not siloed to First Nations health workers but is instead an integral part of the organisational culture, leading to more sustainable improvements in care for First Nations people.

### Study limitations

There was potential for selection bias, as participants who agreed to be interviewed may have had better knowledge or more positive impressions of the FNCC role than participants who declined to be interviewed. Eligibility criteria included those who had worked alongside the FNCC role, so we were unable to capture the perspectives of cancer staff who did not understand the role and its scope. Additionally, while the study aimed to focus on the FNCC role more globally, interviews often referred to the specific individual occupying the role, whom all participants knew personally. This may have inadvertently led participants to conflate the performance of the individual with the broader potential of the role. Importantly, the views of First Nations patients and family members are not represented in this work. These voices are being sought in a further piece of research currently underway.

### Future directions

Additional evaluation is needed to examine the implementation processes and the quantitative impact of FNCC roles on the timeliness of care and adherence to optimal care pathways [41]. As Reilly et al noted, however, the complex and varied tasks of care coordination pose a challenge to the adequate evaluation of these interventions [9]. For instance, while this project highlighted the critical role of relationship building with First Nations communities and individuals, the time commitments and depth of these relationships is challenging to quantify. A combination of qualitative and quantitative evaluation is recommended, with evaluation outcomes determined in partnership with local First Nations communities.

## Conclusions

This study extends our understanding of the scope and impact of IPN/FNCC roles and supports the wider implementation of these roles through Australia. The FNCC role, through trusted relationships with patients, families and healthcare providers, facilitates continuous, culturally informed care that upholds the self-determination of First Nations Australians with cancer.

## Data Availability

Qualitative data from this study are not currently available online.
